# Natural Product Potential of the Genus *Nocardiopsis*

**DOI:** 10.3390/md16050147

**Published:** 2018-04-29

**Authors:** Alyaa Hatem Ibrahim, Samar Yehia Desoukey, Mostafa A. Fouad, Mohamed Salah Kamel, Tobias A. M. Gulder, Usama Ramadan Abdelmohsen

**Affiliations:** 1 Department of Pharmacognosy, Faculty of Pharmacy, Sohag University, Sohag 82524, Egypt; dralyaahatem@gmail.com; 2 Department of Pharmacognosy, Faculty of Pharmacy, Minia University, Minia 61519, Egypt; drsamaryehia@gmail.com (S.Y.D.); m_fouad2000@yahoo.com (M.A.F.); 3 Department of Pharmacognosy, Faculty of Pharmacy, Deraya University, Universities Zone, New Minia City, Minia 61111, Egypt; mskamel2@gmail.com; 4 Department of Chemistry and Center for Integrated Protein Science Munich (CIPSM), Department of Chemistry, Biosystems Chemistry, Technical University of Munich, Lichtenbergstraβe 4, 85748 Garching, Germany

**Keywords:** bioactive, actinomycetes, *Nocardiopsis*, natural products, diversity

## Abstract

Actinomycetes are a relevant source of novel bioactive compounds. One of the pharmaceutically and biotechnologically important genera that attract natural products research is the genus *Nocardiopsis,* mainly for its ability to produce a wide variety of secondary metabolites accounting for its wide range of biological activities. This review covers the literature from January 2015 until February 2018 making a complete survey of all the compounds that were isolated from the genus *Nocardiopsis,* their biological activities, and natural sources, whenever applicable.

## 1. Introduction

Actinobacteria represent one of the largest bacterial phyla, and it is distributed in both terrestrial and marine ecosystems [1,2]. Actinobacteria are prolific producers of a vast variety of bioactive secondary metabolites acting mainly as antiviral, antifungal, anti-trypanosomal, antileishmanial, antimalarial, antibacterial, cytotoxic, antioxidant, and anti-inflammatory drugs [3,4,5,6]. Pharmaceutically active secondary metabolites derived from Actinobacteria account for approximately 70% of the naturally derived compounds that are currently in clinical use [3,4,7]. The first description of the genus *Ncardiopsis* was in 1976 by J. Mayer [8]. *Nocardiopsis* belongs to the order Actinomycetales, family Nocardiopsaceae, and morphologically, it is similar to members of the genera *Actinomadura* and *Nocardia* [8,9]. *Nocardiopsis* species are a Gram-positive, aerobic, halotolerant, and catalase positive actinomycetes. It possesses nocardioform mycelia with long chains of spores on the aerial parts. The genus *Nocardiopsis* is associated with different ecosystems, namely, terrestial as plant epiphytes and endophytes, in addition to marine environments [10].

Bennur et al. made a literature survey on genus *Nocardiopsis* detailing the bioactive compounds isolated prior to 2015 [11]. Reviewing the literature on the genus *Nocardiopsis*, it could produce a wide variety of chemical classes of compounds with diverse biological activities. Secondary metabolites are mainly polyketides [12,13], cyclic peptides [14,15], macrolides [16], diketopiperazines [17,18], α-pyrones [19,20] γ-pyrones [19], alkaloids [21], naphthoquinones [22], phenazines [23], and phenoxazine derivatives [24], which are responsible for a wide spectrum of pharmacological and biological effects , mainly as antibacterial [14], antifungal [25], anticancer [23], antitumour [26], cytotoxic [22,27], immunomodulatory [16], and protein kinase inhibitory [28]. It is also pharmaceutically important for the production of biosurfactants [29]. Within this review, we aim to introduce the most recently discovered chemical diversity that is found in *Nocariopsis*.

**Figure 1:**
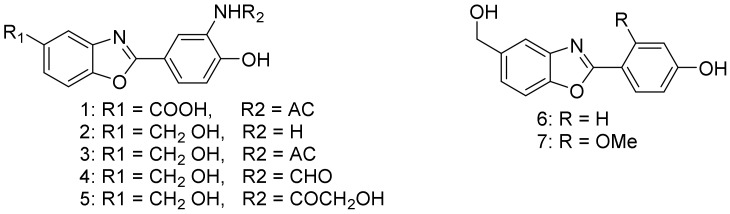
Structural formulas of **1**–**7**.

Nocarbenzoxazoles A−G (**1**−**7**, Figure 1) are new benzoxazole derivatives isolated from *Nocardiopsis lucentensis* DSM 44048. Nocarbenzoxazole G (**7**) showed a cytotoxic activity against HepG2 and HeLa with IC_50_ (half maximal inhibitory concentration, concentration causing 50% of the desired activity) values of 3 and 1 μM, respectively. *Nocardiopsis lucentensis* DSM 44048 is a halophilic strain that was obtained from a soil sample [30].

**Figure 2:**
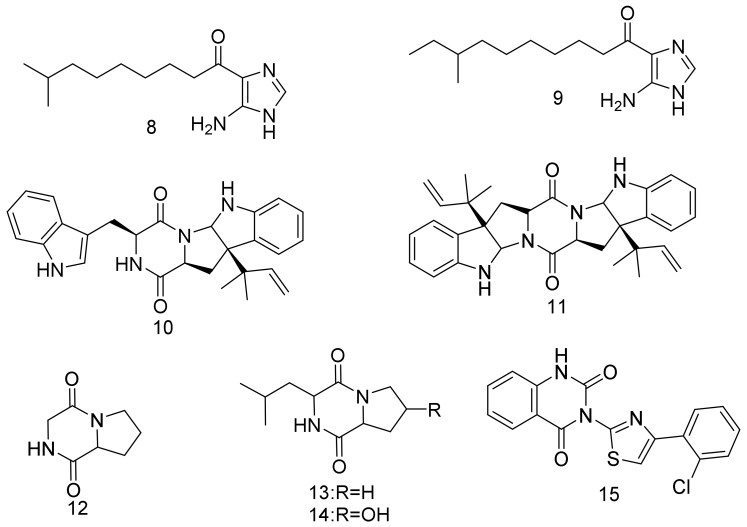
Structural formulas of **8**–**15**.

Chemical investigation of *Nocardiopsis* sp. (strain CNQ115) obtained from marine sediments collected from southern California coasts led to the isolation of nocarimidazoles A (**8**) and B (**9**) as two new 4-aminoimidazole alkaloids. Nocarimidazole A (**8**) showed weak antibacterial activities against *Bacillus subtilis* and *Staphylococcus epidermidis*, with MIC (minimum inhibitory concentration) values of 64 μg/mL [31]. Two diketopiperazines, (**10**) and (**11**), were isolated as SOAT2 (acyl-CoA:cholesterolacyltransferase (ACAT), EC 2.4.1.26) inhibitors from *Nocardiopsis* sp. KM2-16. The study on the cell-based assay showed that (**11)** selectively inhibited SOAT2 activity with a SI (selective index) value of 62. The strain *Nocardiopsis* sp. KM2-16 was isolated in 2012 from sea sediments collected off the coasts of Iriomote Island, Okinawa, Japan [32]. Three diketopiperazines (**12**–**14**) were isolated from *Nocardiopsis umidischolae* KMM 7036. Compound **13** had the strongest cytotoxicity on OSS (the fertility of spermatozoids) test and on the development of embryos of sea urchin (*Strongylocentrotus intermedius)*. At the early blastula stage (5 h), compounds **12–14** did not affect fertilization and embryo development at a concentration of 100 µg/mL. Nevertheless, storage of the embryos in the presence of these compounds (up to 20 h) blocked the embryo development. This led to cell death and embryos lysis, mainly for **(13)**. Compounds **12** and **14** showed cytotoxic activity against splenocytes [33]. A quinazoline derivative identified as 3-[40-(2″-chlorophenyl)-20-thiazolyl]-2,4-dioxo-1,2,3,4-tetrahydroquinazoline (**15**) was isolated from soil-derived *Nocardiopsis alba* that was collected from Western Ghat hills. The compound inhibited telomerases in a Telomeric Repeat Amplification Protocol (TRAP assay) in a cell-free system. Telomerase inhibitory activity was inferred from the reduction in the intensity of the telomerase-elongated TS primers, as compared to the band intensity of internal assay standard (TSNT). Compound **15** was thus suggested to be a suitable molecule for developing a future anticancer drug due to its selectivity towards G-quadruplex over normal DNA duplex and showing lower cytotoxicity for normal cells [34]. Structures are shown in Figure 2.

**Figure 3:**
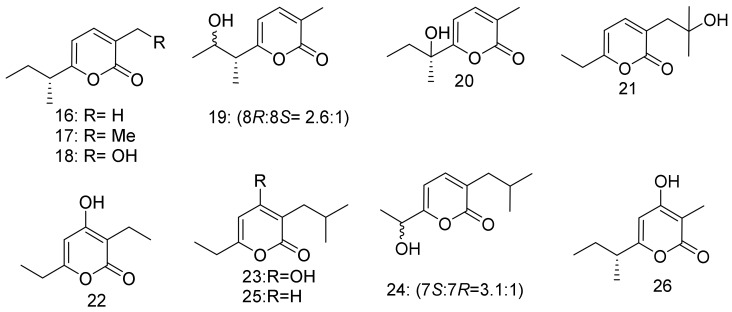
Structural formulas of **16**–**26**.

Eleven α-pyrones **16**–**26** (Figure 3) were isolated from three *Nocardiopsis* strains namely, SCSIO 04583, SCSIO 10419, and SCSIO KS107. Compounds **17**, **19**, **21**, **24**, and **25** displayed a moderate growth inhibition against *Micrococcus luteus,* while **16** and **25** showed mild inhibition against *Bacillus subtilis*. The marine- derived *Nocardiopsis* strains were cultivated from the reef sediment sample that was obtained from Xieyang Island, Beihai, Guangxi Province, China [35].

**Figure 4:**
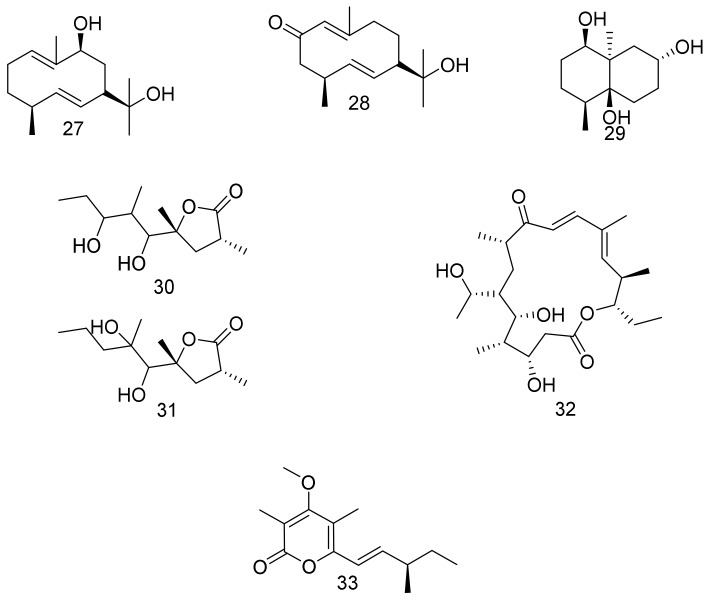
Structural formulas of **27**–**33**.

9*β*-hydroxyl germacradienol (1(10)*E*,5*E*-germacradiene-9*β*,11-diol) (**27**) and 2-oxygermacradienol (11-hydroxy-1(10)*E*,5*E*-germacradien-2-one) (**28**) are two germacradiene-type sesquiterpenes isolated from *Nocardiopsis chromatogenes* YIM 90109 together with (1*β*,4*β*,4a*β*,7*α*,8a*α*)-4,8a-dimethyloctahydronaphthalene-1,4a,7(2H)-triol (**29**) geosmin-type sesquiterpene. *Nocardiopsis chromatogenes* YIM 90109 is a halophilic strain obtained from a saline soil sample collected from Xinjiang Province, China. Compounds **27**–**29** were inactive as antibacterial in paper disc diffusion assay against the tested microbes at 30 μg/disc [36]. Lucentides A (**30**) and B (**31**) are polyketides that were isolated from *Nocardiopsis lucentensis* DSM 44048, together with 19-hydroxyprotylonolide (**32**) all of which had no antimicrobial activities against the tested strains [37]. Marinopyrone D (**33**) was identified from *Nocardiopsis* sp. strain CNQ-675, cultivated from the marine sediment obtained from La Jolla, California, and found to inhibit NO (nitric oxide) production with IC_50_ value of 13 µM. On the other hand, compound **33** had no significant antibacterial activity against a panel of tested Gram-negative and Gram-positive strains. Furthermore, compound **33** at concentrations up to 100 µM was nontoxic to the human renal carcinoma cell line (A498) and the human pancreatic cell lines (MIA-PaCa and PANC-1) [38]. Structures are shown in Figure 4.

**Figure 5:**
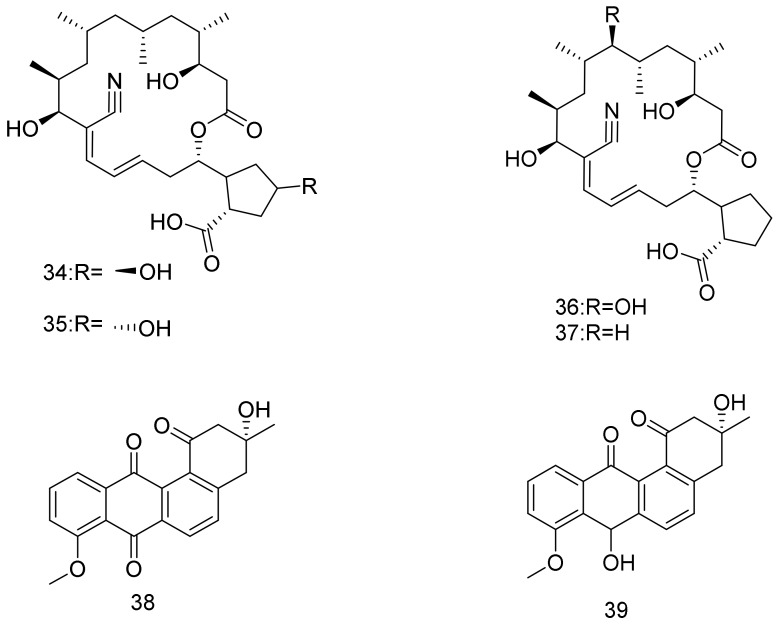
Structural formulas of **34**–**39**.

Borrelidins C–E (**34**–**36**), new 18-membered macrolides along with a previously reported borrelidin (**37**) were isolated from halophilic *Nocardiopsis* strain. Belonging to the rare borrelidin class of antibiotics, borrelidins C (**34**) and D (**35**) showed antibacterial activity against *Salmonella enterica* ATCC 14028, which is a Gram-negative pathogen with MIC values of 16 μM and 63 μM, respectively. Borrelidin C (**34**) displayed cytotoxic activity against SNU638 and K562 cell lines with IC_50_ values of 5.5 μM and 5.7 μM, respectively. Borrelidin D (**35**) showed comparable cytotoxicity against SNU638 and K562 with IC_50_ values of 8.7 μM and 6.7 μM, respectively, whereas borrelidin E (**36**) did not display cytotoxic activity against any of the tested cancer cell lines. The halotolerant *Nocardiopsis* strain was obtained from Jeung-do Island in Shinan-gun, Jeollanamdo, Korea from a hypersaline saltern topsoil samples [39]. A halophilic *Nocardiopsis* strain HR-4 was isolated from a soil sample that was collected from a salt lake in Algerian Sahara. 16S rDNA gene sequence analysis strongly suggests that *Nocardiopsis* strain HR-4 represents novel species. Bioactivity tests with extracts of strain HR-4 showed an antibacterial activity against namely *S. aureus*, *E. faecalis*, and *M. luteus*, as well as antifungal properties against *S. cerevisiae* and *C. albicans*. Chemical investigations led to the isolation and identification of two major angucyclinones, namely, 8-*O*-methyltetrangomycin (**38**) and (−)-7-deoxy-8-*O*-methyltetrangomycin (**39**) [40]. Structures are shown in Figure 5.

**Figure 6:**
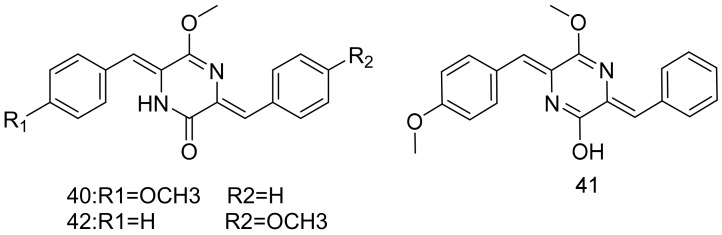
Structural formulas of **40**–**42**.

A cytotoxic compound isomethoxyneihumicin (**40/41**, Figure 6) that was isolated as a mixture of lactam-lactim tautomers together with methoxyneihumicin (**42**) was obtained from *Nocardiopsis alba* KM6-1. Isomethoxyneihumicin (**40/41**) and methoxyneihumicin (**42**) arrested the cell cycle of Jurkat cells in 12 h at the G2/M phase (66 and 67%) at concentrations of 15.0 μM. Both (**40**/**41**) and **42** displayed a cytotoxic activity in 20 h against Jurkat cells with IC_50_ values of 6.98 and 30.5 μM, respectively. These findings suggest that the exhibited cytotoxicity against Jurkat cells is by inhibiting the cell cycle at the G2/M phase. *Nocardiopsis alba* KM6-1 was isolated from the sea sediment samples from Chichijima, Ogasawara, Japan in 2013 [41].

Nocapyrones O-S (**44**–**48**), along with nocapyrone F (**43**), are α-pyrones that were isolated from *Nocardiopsis* sp. YIMM13066 cultured from deep-sea sediment samples. None of the isolated α-pyrones showed any significant cytotoxic activity at 50 μM against PC3, H1299, HeLa, MCF-7, HL7702, and U251 cell lines [42]. 

**Figure 7:**
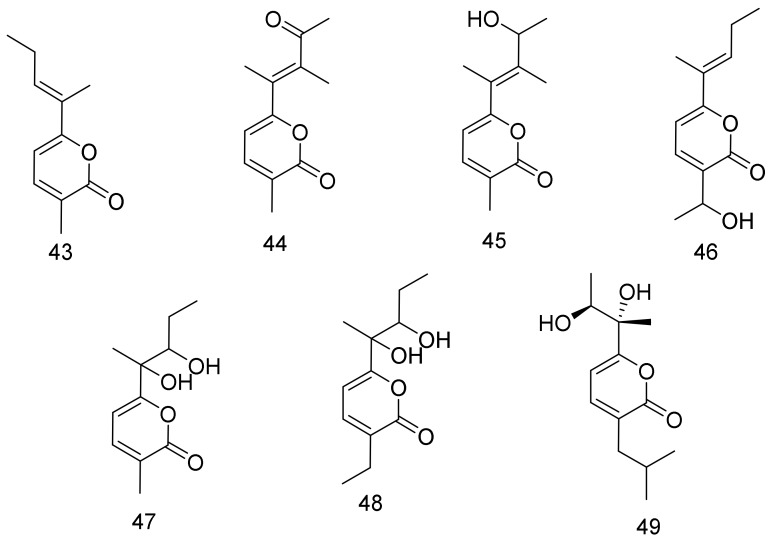
Structural formulas of **43**–**49**.

A new α-pyrone (**49**) was isolated from the deep-sea (Arctic Ocean) actinomycete *Nocardiopsis dassonvillei* subsp. *Dassonvillei* DSM 43111(T) together with five known compounds. Compound **49** did not show any cytotoxicity against MCF-7, K562, A375, SGC7901, HepG2, and Hela cell lines. The five known compounds were identified as cyclo-(L-Pro-L-Val), cyclo-(L-Pro-L-Leu), cyclo-(L-Pro-L-Ile), *N*-(2-hydroxyphenyl)-acetamide, and (4-aminophenyl) acetic acid [43]. Structures are shown in Figure 7.

**Figure 8:**
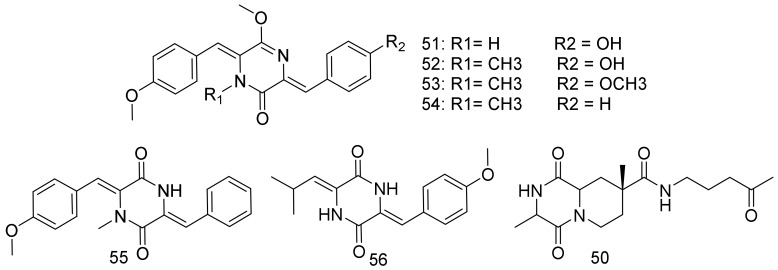
Structural formulas of **50**–**56**.

Nocarazepine A (**50**), which is a diketopiperazine, was purified from *Nocardiopsis alba,* which was cultured from *Anthogorgia caerulea,* that was obtained from the coast of Xieyang Island, Guangxi Province [44]. Six diketopiperazines, of which, two new nocazines F (**51**) and G (**52**), and four known (**53**–**56**) were identified from the deep-sea sediment derived *Nocardiopsis* sp. YIM M13066. Compounds **51** and **52** showed a broad spectrum and excellent cytotoxicity against human cancer cell lines PC3, H1299, HL7702, HeLa, MCF-7, and U251. The most potent was (**52**) against H1299 with IC_50_ 2.60 μM and **51** against H1299 and MCF-7 with IC_50_ 3.87 μM and 3.86 μM, respectively. Only **(52)** showed moderate antibacterial activity against *B. subtilis* ATCC 6051, with an MIC of 25.8 μM. Compounds **51**–**56** were assayed for inhibitory activity against T3SS (type three secretion system) of *S. enteric* serovar Typhimurium UK-1 χ8956. All of the compounds exhibited inhibitory activity at concentration of 100 μM against the secretion of SPI-1 effector SipA/B/C/D and no effects on FliC. Among them, compound **51** exhibited the strongest dose-dependent inhibition on the secretion of the SPI-1 effector SipC (from 5 to 100 μM) [45]. Structures are shown in Figure 8.

*N*-Salicyloyl-2-aminopropan-1,3-diol (**58**), together with a rare aziridine derivative, madurastatin B3 (**57**), were isolated from *Nocardiopsis* sp. LS150010, and both of them exhibited significant antibacterial activity against *S. aureus* and MRSA (methicillin resistant *S. aureus*), with MIC values of 6.25 μg/mL. Compound **57** showed potent antibacterial activity against *Escherichia coli* and *Bacillus subtilis* in addition to potent anti-tuberculosis activity using a BCG-infected THP-1 cell model assay. It showed a significant inhibition of BCG in THP-1 cells growth down to concentrations of 0.156 μg/mL. Remarkably, the active concentration in the infected model was much lower than the MIC value in the cell-based model, which indicates that it may target the pathogen-host interaction. The strain LS150010 was cultured from the soil sample from Sejila Mountain Virgin Forest (altitude 3900 m), southeastern Tibet, China [46].

3′-hydroxy-*N*-(2-oxo-2,5-dihydrofuran-4-yl) propionamide (**59**) and 4-methoxy-2*H*-isoquinolin-1-one (**60**) were isolated together with eight known compounds from *Nocardiopsis lucentensis* sp. ASMR2, which is a new marine strain that was identified from marine plants that were collected from the Red Sea coasts, Hurghada, Egypt. The known compounds were identified as cyclo-(Tyr, Pro), adenosine, indole-3-acetic acid, indole-3-acetic acid methyl ester, indole-3-carboxylic acid, furan-2,5-dimethanol, tyrosol, and glycerol linoleate. Both the filtrate and the cell extracts of *Nocardiopsis lucentensis* sp. ASMR2 displayed moderate antibacterial activity against Gram-positive bacteria (8–12 mm). Interestingly, the extracts of *Nocardiopsis lucentensis* sp. ASMR2 cultivated on solid rice medium exhibited prominent inhibitory activity against *C. albicans* (14 mm), moderate activity (9–11 mm) against *S. cerevisiae*, *B.subtilis*, and *S. aureus*. However, the compounds **59** and **60** exhibited no inhibitory activity. The strain extracts and the produced compounds **59**, **60** displayed no cytotoxicity against the human cervix carcinoma cell line (KB-3-1) and its multidrug-resistant subclone (KBV1) [47].

**Figure 9:**
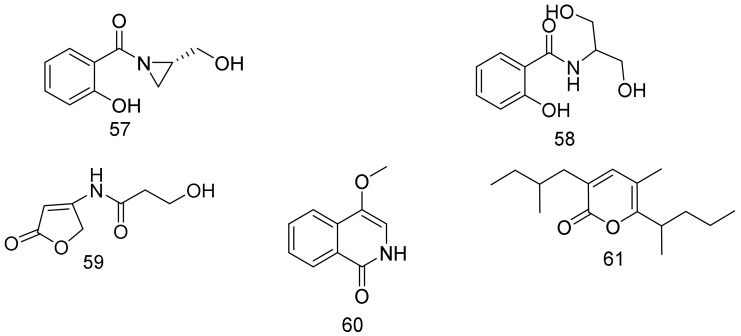
Structural formulas of **57**–**61**.

*Nocardiopsis sp*. NHF48 was isolated from the South China Sea sediments with anti-MRSA activity (MIC value: 12.5 µg/mL). Its chemical investigation led to the isolation of new α-pyrone (**61**) compound with anti-tumor (mouse melanoma cell line) activity (a GI_50_ value of 61.7 µg/mL) [48]. Structures are shown in Figure 9.

**Figure 10:**
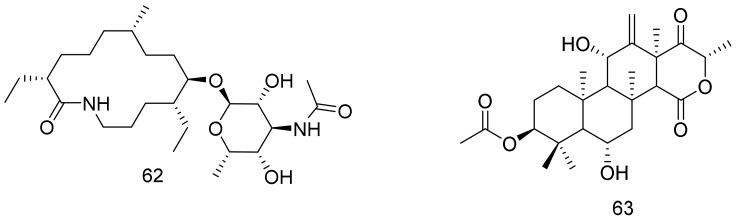
Structural formulas of **62** and **63**.

A 14-membered macrolactam compound, fluvirucin B6 (**62**)**,** was isolated from a marine-derived *Nocardiopsis* sp. CNQ-115. fluvirucin B6 (**62**) showed a weak growth inhibitory activity against Gram-positive bacteria i.e., *Bacillus subtillis* ATCC 6644, *Kocuria rhizophila* ATCC 9341, and *Staphylococcus aureus* ATCC 6538 P with MIC values of 64, 32, and 32 μg/L, respectively. Structures are shown in Figure 10. Whereas, it was inactive against the tested Gram-negative bacteria i.e., *Salmonella typhimurium* ATCC 14028, *Escherichia coli* ATCC 11775, and *Klebsiela pneumoniae* ATCC 4352 up to 128 μg/L [49]. Terretonin N (**63**), a rare tetracyclic 6-hydroxymeroterpenoid, was isolated from the ethyl acetate extract of a solid culture of *Nocardiopsis* sp. together with seven already known compounds i.e., 3-indole acetic acid, anthranilic acid, *N*β-acetyltryptamine, terrain, (3*R*,4*R*)-3,4-dihydroxy-3-methyl-pentan-2-one, (3*S*,4*R*)-3,4-dihydroxy-3-methyl-pentan-2-one, and sitosteryl-3β-d-glucoside . The strain *Nocardiopsis* sp. LGO5 was cultured from the sediment sample that was collected from a lake in Helwan, Egypt. *Nocardiopsis* sp. LGO5 extract displayed pronounced inhibitory activity against *P. aeruginosa* and *S. cerevisiae*; (13 and 14 mm, respectively). It showed low to moderate inhibition against *E. coli* DSMZ 1058, *B. subtilis* DSMZ 704, *P. agarici* DSMZ 11810, *S. warneri* DSMZ 20036, *M. luteus* DSMZ 1605, *S. aureus*, and *C. albicans*. Terretonin N (**63**) displayed distinct antibacterial activity (15mm) against Gram positive *S. warneri*, however, it showed only low activity (7–8mm) against the Gram negative *P. agaraci* and *E. coli*. Both terretonin N (**63**) and the bacterial extract did not show any significant in vitro cytotoxic activity against the human cervix carcinoma cell line KB-3-1 [50].

## 2. Conclusions

**Figure 11:**
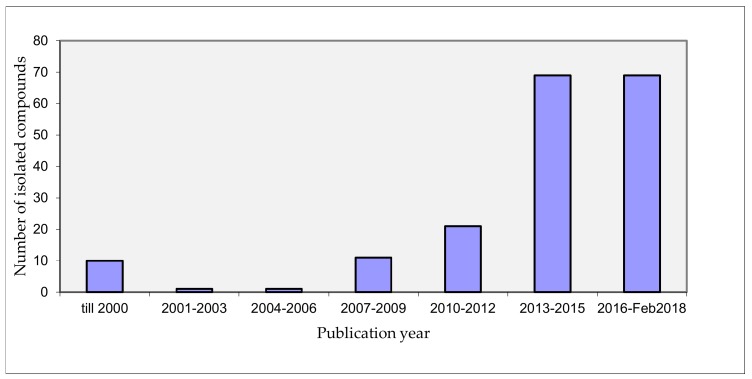
Natural products isolated from genus *Nocardiopsis* according to the year of publication.

The genus *Nocardiopsis* has a remarkable potential to provide therapeutic leads with diverse chemotypes and biological effects. The research on the genus has greatly progressed the recent years (Figure 11).

**Figure 12:**
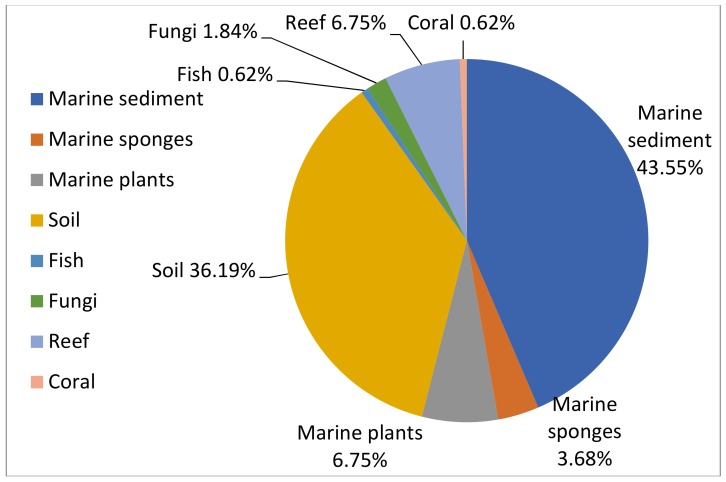
Percentage distribution of natural products from genus *Nocardiopsis*.

It is worth mentioning that the wide occurrence of the genus in diverse habitats (Figure 12) gives great potential for a broad structural, and thus functional, variety of the encoded secondary metabolites. While bioactive compounds were isolated from strains of both marine and terrestrial environments, marine sediments were the richest source of natural products (Figure 12). Almost 22 sequenced genomes from the genus *Nocardiopsis* are deposited so far in NCBI from various marine and terrestrial habitats [51,52,53,54]. The genomic screening and chemical investigation by chromatographic ways is the optimal approach for the prediction and rapid discovery of novel secondary metabolites from microbial strains, such as *Nocardiopsis*.

The genus *Nocardiopsis* has the potential to produce a great diversity of bioactive secondary metabolites, which are promising mainly as cytotoxic, anticancer, antibacterial, and antifungal drugs. Our literature survey showed the most frequently isolated class of compounds from different *Nocardiopsis* species are α-pyrones and diketopiperazine derivatives. The discovery of new species from the genus *Nocardiopsis* indicates that there is great potential for the isolation of new natural products. The genus *Nocardiopsis* harbors a vast collection of yet undiscovered natural products that potentially have a wide range of pharmacological activities, so more effort should be directed towards the investigation of the genus *Nocardiopsis* as an important source for the discovery of novel bioactive natural products for the development of new medications.

## Supplementary Materials

The supplementary data are available online at http://www.mdpi.com/1660-3397/16/5/147/s1.
