# Supplementary material for: Natural Product Potential of the Genus Nocardiopsis
Source: Mar Drugs. 2018 Apr 29;16(5):147. doi: 10.3390/md16050147 (PMC5983278; doi:10.3390/md16050147)
Supplement: Supplementary file 1 [file marinedrugs-16-00147-s001.pdf]

Supplementary data

Compound number	InChI	InChiKeys
**1**	InChI=1S/C16H12N2O6/c1-8(19)24-18-11-6-9(2-4-13(11)20)15-17-12-7-10(16(21)22)3-5-14(12)23-15/h2-7,18,20H,1H3,(H,21,22)	AJYUYIZKZZSUTQ-UHFFFAOYSA-N
**2**	InChI=1S/C14H12N2O3/c15-10-6-9(2-3-12(10)18)14-16-11-5-8(7-17)1-4-13(11)19-14/h1-6,17-18H,7,15H2	QDKBOYBNGQTAPK-UHFFFAOYSA-N
**3**	InChI=1S/C16H14N2O5/c1-9(20)23-18-12-7-11(3-4-14(12)21)16-17-13-6-10(8-19)2-5-15(13)22-16/h2-7,18-19,21H,8H2,1H3	DLEOXPYODXXMQL-UHFFFAOYSA-N
**4**	InChI=1S/C15H11NO4/c17-7-9-1-4-14-12(5-9)16-15(20-14)10-2-3-13(19)11(6-10)8-18/h1-6,8,17,19H,7H2	SQJFTGHIDYFTID-UHFFFAOYSA-N
**5**	InChI=1S/C16H13NO5/c18-7-9-1-4-15-12(5-9)17-16(22-15)10-2-3-13(20)11(6-10)14(21)8-19/h1-6,18-20H,7-8H2	MVGWDWHBEAGUKI-UHFFFAOYSA-N
**6**	InChI=1S/C14H11NO3/c16-8-9-1-6-13-12(7-9)15-14(18-13)10-2-4-11(17)5-3-10/h1-7,16-17H,8H2	CSCMODQTLPIEMH-UHFFFAOYSA-N
**7**	InChI=1S/C15H13NO4/c1-19-14-7-10(18)3-4-11(14)15-16-12-6-9(8-17)2-5-13(12)20-15/h2-7,17-18H,8H2,1H3	FMKKXSLFSSVDCS-UHFFFAOYSA-N
**8**	InChI=1S/C13H23N3O/c1-10(2)7-5-3-4-6-8-11(17)12-13(14)16-9-15-12/h9-10H,3-8,14H2,1-2H3,(H,15,16)	WMNFVUHNJUDHER-UHFFFAOYSA-N
**9**	InChI=1S/C14H25N3O/c1-3-11(2)8-6-4-5-7-9-12(18)13-14(15)17-10-16-13/h10-11H,3-9,15H2,1-2H3,(H,16,17)	OPDPHQGPKVQMGO-UHFFFAOYSA-N
**10**	InChI=1S/C27H28N4O2/c1-4-26(2,3)27-14-22-23(32)29-21(13-16-15-28-19-11-7-5-9-17(16)19)24(33)31(22)25(27)30-20-12-8-6-10-18(20)27/h4-12,15,21-22,25,28,30H,1,13-14H2,2-3H3,(H,29,32)/t21-,22-,25?,27+/m0/s1	VBYPRAJLFYXBBP-PBJMKJCMSA-N
**11**	InChI=1S/C32H36N4O2/c1-7-29(3,4)31-17-23-25(37)36-24(26(38)35(23)27(31)33-21-15-11-9-13-19(21)31)18-32(30(5,6)8-2)20-14-10-12-16-22(20)34-28(32)36/h7-16,23-24,27-28,33-34H,1-2,17-18H2,3-6H3/t23-,24+,27?,28?,31+,32-	VKEAHNPKYMHYJJ-LAKZOROHSA-N
**12**	InChI=1S/C7H10N2O2/c10-6-4-8-7(11)5-2-1-3-9(5)6/h5H,1-4H2,(H,8,11)	OWOHLURDBZHNGG-UHFFFAOYSA-N
**13**	InChI=1S/C11H18N2O2/c1-7(2)6-8-11(15)13-5-3-4-9(13)10(14)12-8/h7-9H,3-6H2,1-2H3,(H,12,14)	SZJNCZMRZAUNQT-UHFFFAOYSA-N

**14**	InChI=1S/C11H18N2O3/c1-6(2)3-8-11(16)13-5-7(14)4-9(13)10(15)12-8/h6-9,14H,3-5H2,1-2H3,(H,12,15)	YEHIUWVXPQQDMC-UHFFFAOYSA-N
**15**	InChI=1S/C17H10ClN3O2S/c18-12-7-3-1-5-10(12)14-9-24-17(20-14)21-15(22)11-6-2-4-8-13(11)19-16(21)23/h1-9H,(H,19,23)	XOUVQBQYRLTUIV-UHFFFAOYSA-N
**16**	InChI=1S/C10H14O2/c1-4-7(2)9-6-5-8(3)10(11)12-9/h5-7H,4H2,1-3H3/t7-/m1/s1	MGLYOXCODWYDGM-SSDOTTSWSA-N
**17**	InChI=1S/C11H16O2/c1-4-8(3)10-7-6-9(5-2)11(12)13-10/h6-8H,4-5H2,1-3H3/t8-/m1/s1	PFHBQBOWTMLEJD-MRVPVSSYSA-N
**18**	InChI=1S/C10H14O3/c1-3-7(2)9-5-4-8(6-11)10(12)13-9/h4-5,7,11H,3,6H2,1-2H3/t7-/m1/s1	YGWDSGLEFHLHNC-SSDOTTSWSA-N
**19**	InChI=1S/C10H14O3/c1-6-4-5-9(13-10(6)12)7(2)8(3)11/h4-5,7-8,11H,1-3H3/t7-,8?/m1/s1	SZVFNJKDIZEIMR-GVHYBUMESA-N
**20**	InChI=1S/C10H14O3/c1-4-10(3,12)8-6-5-7(2)9(11)13-8/h5-6,12H,4H2,1-3H3/t10-/m1/s1	FZRIAELUSZPPOS-SNVBAGLBSA-N
**21**	InChI=1S/C11H16O3/c1-4-9-6-5-8(10(12)14-9)7-11(2,3)13/h5-6,13H,4,7H2,1-3H3	YLTBUTSTBPLRGO-UHFFFAOYSA-N
**22**	InChI=1S/C9H12O3/c1-3-6-5-8(10)7(4-2)9(11)12-6/h5,10H,3-4H2,1-2H3	YZKHJSNBJTVBQW-UHFFFAOYSA-N
**23**	InChI=1S/C11H16O3/c1-4-8-6-10(12)9(5-7(2)3)11(13)14-8/h6-7,12H,4-5H2,1-3H3	LLSJXJAHLPCJOQ-UHFFFAOYSA-N
**24**	InChI=1S/C11H16O3/c1-7(2)6-9-4-5-10(8(3)12)14-11(9)13/h4-5,7-8,12H,6H2,1-3H3	SGBVQGMWPRFHKS-UHFFFAOYSA-N
**25**	InChI=1S/C11H16O2/c1-4-10-6-5-9(7-8(2)3)11(12)13-10/h5-6,8H,4,7H2,1-3H3	MKZUGSPKAYXMHH-UHFFFAOYSA-N
**26**	InChI=1S/C10H14O3/c1-4-6(2)9-5-8(11)7(3)10(12)13-9/h5-6,11H,4H2,1-3H3/t6-/m1/s1	QKYRDLBTCVVFJE-ZCFIWIBFSA-N
**27**	InChI=1S/C15H26O2/c1-11-6-5-7-12(2)14(16)10-13(9-8-11)15(3,4)17/h7-9,11,13-14,16-17H,5-6,10H2,1-4H3/b9-8+,12-7-/t11-,13+,14-/m0/s1	DXVZUYIXOHXNBU-ZQZRAIAYSA-N
**28**	InChI=1S/C15H24O2/c1-11-5-7-13(15(3,4)17)8-6-12(2)10-14(16)9-11/h5,7,10-11,13,17H,6,8-9H2,1-4H3/b7-5+,12-10-/t11-,13-/m1/s1	NRZAKJLNYYCGMN-CQTPFTFLSA-N
**29**	InChI=1S/C12H22O3/c1-8-3-4-10(14)11(2)7-9(13)5-6-12(8,11)15/h8-10,13-15H,3-7H2,1-2H3/t8-,9+,10+,11+,12-/m0/s1	TVBXZYIQQHPCFC-KQSJRHEJSA-N
**30**	InChI=1S/C12H22O4/c1-5-9(13)8(3)10(14)12(4)6-	QKUJJZCQQUIM

	7(2)11(15)16-12/h7-10,13-14H,5-6H2,1-4H3/t7-,8?,9?,10?,12-/m1/s1	AA-KRYQXMBOSA-N
**31**	InChI=1S/C12H22O4/c1-5-6-11(3,15)10(14)12(4)7-8(2)9(13)16-12/h8,10,14-15H,5-7H2,1-4H3/t8-,10?,11?,12-/m1/s1	CMRPPBFRISETMO-OZOYIQBDSA-N
**32**	InChI=1S/C23H38O6/c1-7-21-15(4)10-13(2)8-9-19(25)14(3)11-18(17(6)24)23(28)16(5)20(26)12-22(27)29-21/h8-10,14-18,20-21,23-24,26,28H,7,11-12H2,1-6H3/b9-8+,13-10+/t14-,15+,16-,17-,18+,20-,21-,23+/m0/s1	LIMMXDFDIDWIHU-FDDVHUOSSA-N
**33**	InChI=1S/C14H20O3/c1-6-9(2)7-8-12-10(3)13(16-5)11(4)14(15)17-12/h7-9H,6H2,1-5H3/b8-7+/t9-/m1/s1	LEOSHPKVZGISFK-FCZSHJHJSA-N
**34**	InChI=1S/C28H43NO7/c1-16-9-17(2)11-19(4)27(33)20(15-29)7-5-6-8-25(22-12-21(30)13-23(22)28(34)35)36-26(32)14-24(31)18(3)10-16/h5-7,16-19,21-25,27,30-31,33H,8-14H2,1-4H3,(H,34,35)/b6-5+,20-7+/t16-,17-,18+,19+,21-,22?,23+,24-,25+,27+/m1/s1	XPAMOXHOVDBZNA-KQZSSORHSA-N
**35**	InChI=1S/C28H43NO7/c1-16-9-17(2)11-19(4)27(33)20(15-29)7-5-6-8-25(22-12-21(30)13-23(22)28(34)35)36-26(32)14-24(31)18(3)10-16/h5-7,16-19,21-25,27,30-31,33H,8-14H2,1-4H3,(H,34,35)/b6-5+,20-7+/t16-,17-,18+,19+,21+,22?,23+,24-,25+,27+/m1/s1	XPAMOXHOVDBZNA-LGWQXCRGSA-N
**36**	InChI=1S/C27H41NO7/c1-16-11-12-22(29)17(2)13-18(3)23(30)14-25(31)35-24(20-8-6-9-21(20)27(33)34)10-5-4-7-19(15-28)26(16)32/h4-5,7,16-18,20-24,26,29-30,32H,6,8-14H2,1-3H3,(H,33,34)/b5-4+,19-7+/t16-,17-,18-,20?,21-,22+,23+,24-,26-/m0/s1	IKAWPOUWBBICMH-QUJKPQATSA-N
**37**	InChI=1S/C27H41NO6/c1-17-8-6-9-18(2)26(31)20(16-28)10-4-5-13-24(21-11-7-12-22(21)27(32)33)34-25(30)15-23(29)19(3)14-17/h4-5,10,17-19,21-24,26,29,31H,6-9,11-15H2,1-3H3,(H,32,33)/b5-4+,20-10+/t17-,18+,19+,21?,22+,23-,24+,26+/m1/s1	UQJWAODIRWCVRY-FLZVVRJXSA-N
**38**	InChI=1S/C20H16O5/c1-20(24)8-10-6-7-12-17(15(10)13(21)9-20)19(23)11-4-3-5-14(25-2)16(11)18(12)22/h3-7,24H,8-9H2,1-2H3/t20-/m1/s1	XZLGWJORNHETKI-HXUWFJFHSA-N
**39**	InChI=1S/C20H18O5/c1-20(24)8-10-6-7-12-17(15(10)13(21)9-20)19(23)11-4-3-5-14(25-2)16(11)18(12)22/h3-7,18,22,24H,8-9H2,1-2H3/t18?,20-/m1/s1	RHBBTFAZTFDHEG-ROPPNANJSA-N
**40**	InChI=1S/C20H18N2O3/c1-24-16-10-8-15(9-11-16)13-18-20(25-2)22-17(19(23)21-18)12-14-6-4-3-5-7-14/h3-13H,1-2H3,(H,21,23)/b17-12-,18-13-	CYZMZJISYQZNAE-MZGHLJKDSA-N
**41**	InChI=1S/C20H18N2O3/c1-24-16-10-8-15(9-11-16)13-18-20(25-2)22-17(19(23)21-18)12-14-6-4-3-5-7-14/h3-13H,1-2H3,(H,21,23)/b17-12-,18-13-	CYZMZJISYQZNAE-MZGHLJKDSA-N

**42**	InChI=1S/C20H18N2O3/c1-24-16-10-8-15(9-11-16)12-17-19(23)21-18(20(22-17)25-2)13-14-6-4-3-5-7-14/h3-13H,1-2H3,(H,21,23)/b17-12-,18-13-	UQGUUQAUHGSKIS-MZGHLJKDSA-N
**43**	InChI=1S/C11H14O2/c1-4-5-8(2)10-7-6-9(3)11(12)13-10/h5-7H,4H2,1-3H3/b8-5+	PBQULXLDQBOQEV-VMPITWQZSA-N
**44**	InChI=1S/C12H14O3/c1-7-5-6-11(15-12(7)14)9(3)8(2)10(4)13/h5-6H,1-4H3/b9-8+	FCISLHWNONCLCE-CMDGGOBGSA-N
**45**	InChI=1S/C12H16O3/c1-7-5-6-11(15-12(7)14)9(3)8(2)10(4)13/h5-6,10,13H,1-4H3/b9-8+	DPIOXNJRDMNKDX-CMDGGOBGSA-N
**46**	InChI=1S/C12H16O3/c1-4-5-8(2)11-7-6-10(9(3)13)12(14)15-11/h5-7,9,13H,4H2,1-3H3/b8-5+	VSEOEXSPCMUHTQ-VMPITWQZSA-N
**47**	InChI=1S/C11H16O4/c1-4-8(12)11(3,14)9-6-5-7(2)10(13)15-9/h5-6,8,12,14H,4H2,1-3H3	NZUBKBRDWGWBNG-UHFFFAOYSA-N
**48**	InChI=1S/C12H18O4/c1-4-8-6-7-10(16-11(8)14)12(3,15)9(13)5-2/h6-7,9,13,15H,4-5H2,1-3H3	VNKYTWPVKUBULZ-UHFFFAOYSA-N
**49**	InChI=1S/C13H20O4/c1-8(2)7-10-5-6-11(17-12(10)15)13(4,16)9(3)14/h5-6,8-9,14,16H,7H2,1-4H3/t9-,13-/m0/s1	PUHCIPQAMDGUGT-ZANVPECISA-N
**50**	InChI=1S/C16H25N3O4/c1-10(20)5-4-7-17-15(23)16(3)6-8-19-12(9-16)13(21)18-11(2)14(19)22/h11-12H,4-9H2,1-3H3,(H,17,23)(H,18,21)/t11?,12?,16-/m1/s1	SUEITZDEFMXEAW-ZEPSKSRBSA-N
**51**	InChI=1S/C20H18N2O4/c1-25-16-9-5-14(6-10-16)12-18-20(26-2)22-17(19(24)21-18)11-13-3-7-15(23)8-4-13/h3-12,23H,1-2H3,(H,21,24)/b17-11-,18-12-	SNSZPOPKOYKHDP-WHYMJUELSA-N
**52**	InChI=1S/C21H20N2O4/c1-23-19(13-15-6-10-17(26-2)11-7-15)20(27-3)22-18(21(23)25)12-14-4-8-16(24)9-5-14/h4-13,24H,1-3H3/b18-12-,19-13-	HRMZDSPJGINLAD-BKHHGCLFSA-N
**53**	InChI=1S/C22H22N2O4/c1-24-20(14-16-7-11-18(27-3)12-8-16)21(28-4)23-19(22(24)25)13-15-5-9-17(26-2)10-6-15/h5-14H,1-4H3/b19-13-,20-14-	PIZYGUXDBZYKHO-AXPXABNXSA-N
**54**	InChI=1S/C21H20N2O3/c1-23-19(14-16-9-11-17(25-2)12-10-16)20(26-3)22-18(21(23)24)13-15-7-5-4-6-8-15/h4-14H,1-3H3/b18-13-,19-14-	NCBOROGHSUXZPE-SXQSXHJWSA-N
**55**	InChI=1S/C20H18N2O3/c1-22-18(13-15-8-10-16(25-2)11-9-15)19(23)21-17(20(22)24)12-14-6-4-3-5-7-14/h3-13H,1-2H3,(H,21,23)/b17-12-,18-13-	NTIUCMLVFKVNDG-MZGHLJKDSA-N
**56**	InChI=1S/C16H18N2O3/c1-10(2)8-13-15(19)18-14(16(20)17-13)9-11-4-6-12(21-3)7-5-11/h4-10H,1-3H3,(H,17,20)(H,18,19)/b13-8-,14-9-	YRVUIYSTPTWLCY-QKPPEBSVSA-N
57	InChI=1S/C10H11NO3/c12-6-7-5-11(7)10(14)8-3-1-2-4-9(8)13/h1-4,7,12-13H,5-6H2/t7-,11?/m0/s1	WQZHSQZSDADXLF-

		RGENBBCFSA-N
58	InChI=1S/C10H13NO4/c12-5-7(6-13)11-10(15)8-3-1-2-4-9(8)14/h1-4,7,12-14H,5-6H2,(H,11,15)	DQQQQCYBNYTEEO-UHFFFAOYSA-N
59	InChI=1S/C7H9NO4/c9-2-1-6(10)8-5-3-7(11)12-4-5/h3,9H,1-2,4H2,(H,8,10)	FYESINSTVTVKDJ-UHFFFAOYSA-N
60	InChI=1S/C10H9NO2/c1-13-9-6-11-10(12)8-5-3-2-4-7(8)9/h2-6H,1H3,(H,11,12)	ITVVDKFBWYVGPV-UHFFFAOYSA-N
61	InChI=1S/C16H26O2/c1-6-8-12(4)15-13(5)10-14(16(17)18-15)9-11(3)7-2/h10-12H,6-9H2,1-5H3	JTEPOCODFJKCCN-UHFFFAOYSA-N
62	InChI=1S/C26H48N2O6/c1-6-19-12-9-15-27-25(32)20(7-2)11-8-10-16(3)13-14-21(19)34-26-24(31)22(28-18(5)29)23(30)17(4)33-26/h16-17,19-24,26,30-31H,6-15H2,1-5H3,(H,27,32)(H,28,29)/t16-,17-,19+,20+,21+,22-,23+,24+,26-/m0/s1	WEVZGKSDDNCUFL-WOTYRYJGSA-N
63	InChI=1S/C26H38O7/c1-12-17(29)19-24(6)10-9-16(33-14(3)27)23(4,5)18(24)15(28)11-25(19,7)20-22(31)32-13(2)21(30)26(12,20)8/h13,15-20,28-29H,1,9-11H2,2-8H3/t13-,15-,16-,17+,18?,19?,20?,24+,25-,26+/m0/s1	ZCWNEJRDYFTTHU-NPJNTPFJSA-N
